# Präoperative MRT-Bildgebung bei Hüftdysplasie: Abklärung assoziierter Deformitäten und intraartikulärer Pathologien

**DOI:** 10.1007/s00132-023-04356-8

**Published:** 2023-03-28

**Authors:** Till D. Lerch, Florian Schmaranzer

**Affiliations:** Universitätsinstitut für diagnostische, interventionelle und pädiatrische Radiologie, Inselspital Bern, Universität Bern, Freiburgstr. 8, 3010 Bern, Schweiz

**Keywords:** Femorale antetorsion, Knorpel, Femoroazetabuläres Impingement, Hüftdysplasie, Magnetresonanztomographie, Femoral antetorsion, Cartilage, Femoracetabular impingement, Hip dysplasia, Magnetic resonance imaging

## Abstract

**Hintergrund:**

Die Hüftdysplasie ist ein bekannter Grund für Hüftschmerzen bei Jugendlichen und jungen Erwachsenen. Die präoperative Bildgebung wurde durch die Fortschritte der MRT in den letzten Jahren immer wichtiger und hat heute einen hohen Stellenwert.

**Ziel der Arbeit:**

Dieser Artikel soll einen Überblick über die präoperative Bildgebung bei Hüftdysplasie geben. Die azetabuläre Version, assoziierte femorale Deformitäten (Cam-Deformität, Valgus und femorale Antetorsion) und intraartikuläre Pathologien (Labrum- und Knorpelschäden) sowie verschiedene Messmethoden und Normwerte werden beschrieben.

**Methoden:**

Die präoperative Bildgebung bei Hüftdysplasie beginnt mit dem a. p. Becken-Röntgenbild.

Die Schnittbildgebung (CT oder MRT) ist der Goldstandard zur Beurteilung der azetabulären Version, der Cam-Deformität und zur Messung der femoralen Antetorsion. Die azetabuläre Version und Überdachung sowie assoziierte femorale Deformitäten können detailliert analysiert werden. Für die femorale Antetorsion sollten die verschiedenen Messmethoden und Normalwerte berücksichtigt werden. Diese sind vor allem bei Patienten mit erhöhter femoraler Antetorsion zu beachten. Die MRT ermöglicht die Beurteilung der Labrumhypertrophie und subtile Zeichen der Hüftinstabilität. 3‑D-Knorpelmapping mittels MRT ermöglicht eine Quantifizierung der Knorpeldegeneration. Ossäre 3D Modelle mittels 3D‑CT und zunehmend mittels MRT ermöglichen zudem die Detektion extraartikulärer Pathologien, (z. B. ischiofemorales Impingement) die als Differenzialdiagnosen zu berücksichtigen sind.

**Ergebnisse und Diskussion:**

Die Hüftdysplasie kann in anteriore, laterale und posteriore Dysplasie eingeteilt werden. Kombinierte Deformitäten sind bei der Hüftdysplasie häufig, eine Cam-Deformität kann bei bis zu 86 % der Patienten vorliegen. Valgusdeformitäten wurden bei 44 % beschrieben. Eine Hüftdysplasie kombiniert mit erhöhter femoraler Antetorsion kann bei bis zu 52 % der Patienten vorliegen. Bei erhöhter femoraler Antetorsion kann zudem ein posteriores intra- und extraartikuläres (ischiofemorales) Impingement zwischen dem Trochanter minor und dem Tuber ischiadicum auftreten. Labrumläsionen und -hypertrophie, subchondrale Zysten und Knorpelschäden sind typische Kollateralschäden bei Patienten mit Hüftdysplasie. Die Hypertrophie des M. iliocapsularis ist ein Instabilitäts Zeichen. Bei der präoperativen Bildgebung sind sowohl die azetabuläre Version und Morphologie als auch die femoralen Deformitäten zu berücksichtigen. Labrum- und Knorpelschäden sowie die assoziierten Deformitäten (u. a. Cam-Deformität und femorale Antetorsion) sind wichtig für die Therapieplanung von Patienten mit Hüftdysplasie. Die biochemische MRT-Bildgebung mittels Knorpelmapping-Techniken (u. a. dGEMRIC) hat großes Potenzial die präoperative Diagnostik und die Patientenselektion zu verbessern.

## Abstract (en)

**Background:**

Developmental dysplasia of the hip (DDH) is a known reason for hip pain for adolescents and young adults. Preoperative imaging is increasingly recognized as an important factor due to the recent advances in MR imaging.

**Objectives:**

The aim of this article is to give an overview of preoperative imaging for DDH. The acetabular version and morphology, associated femoral deformities (cam deformity, valgus and femoral antetorsion) and intraarticular pathologies (labrum and cartilage damage) and cartilage mapping are described.

**Methods:**

After an initial evaluation with AP radiographs, CT or MRI represent the methods of choice for the preoperative evaluation of the acetabular morphology and cam deformity, and for the measurement of femoral torsion. Different measurement techniques and normal values should be considered, especially for patients with increased femoral antetorsion because this could lead to misinterpretation and misdiagnosis. MRI allows analysis of labrum hypertrophy and subtle signs for hip instability. 3D MRI for cartilage mapping allows quantification of biochemical cartilage degeneration and yields great potential for surgical decision-making. 3D-CT and, increasingly, 3D MRI of the hip to generate 3D pelvic bone models and subsequent 3D impingement simulation can help to detect posterior extraarticular ischiofemoral impingement.

**Results and discussion:**

Acetabular morphology can be divided in anterior, lateral and posterior hip dysplasia. Combined osseous deformities are common, such as hip dysplasia combined with cam deformity (86%). Valgus deformities were reported in 44%. Combined hip dysplasia and increased femoral antetorsion can occur in 52%. Posterior extraarticular ischiofemoral impingement between the lesser trochanter and the ischial tuberosity can occur in patients with increased femoral antetorsion. Typically, labrum damage and hypertrophy, cartilage damage, subchondral cysts can occur in hip dysplasia. Hypertrophy of the muscle iliocapsularis is a sign for hip instability. Acetabular morphology and femoral deformities (cam deformity and femoral anteversion) should be evaluated before surgical therapy for patients with hip dysplasia, considering the different measurement techniques and normal values of femoral antetorsion.

Die präoperative Bildgebung bei Hüftdysplasie zur Analyse der Morphologie der Hüftpfanne und des proximalen Oberschenkels sowie der damit verbundenen Deformitäten (cam-deformität, Valgus und femorale Antetorsion) und intraartikulären Pathologien (Labrum- und Knorpelschäden) sowie Knorpelmapping ist notwendig, um eine adäquate Evaluation des Gelenks zu gewährleisten.

## Einleitung

Die Hüftdysplasie ist definiert als verminderte Überdachung (anterior oder lateral) des Femurkopfes und als das Vorliegen einer zu steil gestellten Hüftpfanne. Das Grundproblem ist die verkleinerte Facies lunata bei Patienten mit Hüftdysplasie [64]. Pathophysiologisch führen die lateralen Druckspitzen auf Knorpel und Labrum durch die statische Überbelastung am Pfannenrand zu einer lateral betonten Hüftgelenksarthrose [44]. Die Beckenform bei Hüftdysplasie erscheint typischerweise viereckig oder rektangulär und die Beckenschaufel innenrotiert, während das Tuber ischiadicum außenrotiert erscheint [35, 74]. Die Tendenz des Femurkopfs zur Migration nach superolateral und die resultierende Instabilität können bei Patienten mit einer Coxa valga et antetorta (Valgusdeformität und erhöhte femorale Antetorsion) zusätzlich verstärkt werden kann.

Verschiedene chirurgische Therapien zur Behandlung der Hüftdysplasie wurden beschrieben. Das gemeinsame biomechanische Ziel der Beckenosteotomien ist eine Reduktion der pathologisch erhöhten lateralen Druckspitzen der dysplastischen Hüften [62]. Beckenosteotomien, allen voran die periazetabuläre Osteotomie (PAO), ermöglichen die korrigierende Reorientierung des dysplastischen Azetabulums zur Verbesserung der Überdachung des Femurkopfs. Dadurch können in der Mehrheit der Fälle die Gelenkfunktion wiederhergestellt, die Hüftschmerzen reduziert und langfristig das native Hüftgelenk erhalten werden [33].

## Ziel der Arbeit

Dieser Artikel soll einen Überblick über die präoperative Bildgebung bei Hüftdysplasie geben. Die azetabuläre Version und femorale Deformitäten, die Cam-Deformität, Labrum und Knorpel-Bildgebung sowie die verschiedenen Messmethoden und Normwerte der femoralen Antetorsion werden beschrieben.

## Methoden

Die präoperative Bildgebung bei Hüftdysplasie beginnt mit der a.-p. (anteroposterior) Beckenübersicht als Röntgenaufnahme. Diese werden im ersten Artikel in dieser Ausgabe ausführlich besprochen. Die wichtigsten Parameter sind der lateraler Zentrum-Eck-Winkel (LCE-Winkel, „lateral center edge“) und der azetabuläre Index (AI, Synonym: AC-Winkel oder Tragflächenwinkel) (Tab. 1). Definiert wird die Hüftdysplasie als LCE-Winkel von weniger als 18° (Tab. 1) von Tönnis und Kollegen [77]. Ein LCE-Winkel zwischen 20 und 25° definiert die grenzwertige Hüftdysplasie (Borderline-Hüftdysplasie) [41]. Weitere radiologische diagnostische Parameter für die Hüftdysplasie sind der azetabuläre Index > 14° und eine Femurkopfextrusion > 27 % (Tab. 1). Die a.-p. Beckenübersichtsaufnahme wird meistens mit einer lateralen oder axialen Aufnahme des proximalen Femurs ergänzt [72]. Auf dieser kann der anteriore Schenkelhals beurteilt und der Alphawinkel gemessen werden [47]. Dabei hat sich die modifizierte Dunn-Projektion (45° Hüftflexion in maximaler Hüftabduktion, Zentralstrahl auf den Femurkopf ausgerichtet) als sensitivste Aufnahme zur initialen und postoperativen Beurteilung des anterolateralen Schenkelhalses gezeigt [60]. Häufig wird die diagnostische Abklärung ergänzt mit einer Faux-Profil-Aufnahme zur Berechnung des vorderen Pfannendachwinkel (englisch „anterior center edge“). Der Normwert beträgt > 25°, ein Winkel unter 20° ist pathologisch und beschreibt die ventrale Dysplasie.

**Table 1:** 

Parameter	Hüftdysplasie	Interpretation
Radiologische Diagnose Beckenröntgenbild a.-p.	**Lateraler Zentrum-Eck-Winkel <** **20°**	Hüftdysplasie
	**Lateraler Zentrum-Eck-Winkel 20–25°**	Grenzwertige Hüftdysplasie
	Azetabulärer Index > 14°	Hüftdysplasie
	Femurkopfextrusion > 27 %	Hüftdysplasie
	FEAR-Index > 5°	Instabilitätszeichen
	Unterbrochene Shenton-Linie	Instabilitätszeichen
	CCD-Winkel > 139°	Valgusdeformität
MRT axial	**Azetabuläre Morphologie**	Anteriore oder laterale Hüftdysplasie
	**Azetabuläre Anteversion**	Orientierung Azetabulum
	**Alphawinkel >** **55°**	Cam-Deformität
	**Femorale Antetorsion**	Falls erhöht, Cave posteriores Impingement
	**Kombinierte Anteversion**	McKibbin-Index
MRT Muskulatur	Hypertrophie M. iliocapsularis	Instabilitätszeichen
MRT Labrum	Labrumhypertrophie	Statische Überlastung durch defizitäre Überdachung
MRT Knorpel	Knorpelschaden	Wichtig für Entscheidung hüftgelenkserhaltende Operation ja/nein
Arthro-MRT	Kontrastmittel im posterioren Gelenkspalt	Instabilitätszeichen oder Zeichen der Degeneration
Beckenform (Röntgenbild)	Typischerweise innenrotierte Beckenschaufel und außenrotierte Schambeinäste	Im Vergleich zu außenrotierter Beckenschaufel bei azetabulärer Retroversion
3‑D-Modell	Patientenspezifische Impingement-Analyse	Bei erhöhter femoraler Antetorsion und Verdacht auf ischiofemorales Impingement
Zusatzsequenzen MRT	Knorpelmapping Größe Facies lunata	*Bei grenzwertiger Hüftdysplasie*

*CCD* Centrum-Collum-Diaphyse, *FEAR* „femoro-epiphyseal acetabular roof“

Die Beckenübersichtsaufnahme kann liegend oder stehend erfolgen und sollte beckenzentriert erfolgen [72] und nicht hüftzentriert wie zur Planung einer Hüftprothese. Die Beckenübersichtsaufnahme sollte mit innenrotierten Beinen (15°) und mit einem Film-Fokus-Abstand von 1,2 m erfolgen [72]. Dabei sollte auf die Beckenorientierung und -kippung geachtet werden, welche vor allem die Darstellung der azetabulären Version beeinflussen können [73]. Auf der Beckenübersichtsaufnahme kann zudem die superolaterale „Pistol-grip“-Deformität erkannt werden.

Die Tönnis-Klassifikation ermöglicht eine erste Einschätzung der degenerativen Veränderungen auf dem Röntgenbild

Die Tönnis-Klassifikation beruht vor allem auf der Gelenkspaltverschmälerung und ermöglicht eine erste Einschätzung der degenerativen Veränderungen [75]. Alternativ kann die Kellgren-Lawrence-Klassifikation verwendet werden. Bei fortgeschrittener Arthrose (ab Tönnis-Grad 2) oder bei Alter über 40 Jahren hat eine hüftgelenkserhaltende Operation, z. B. eine PAO, zur Behandlung einer Hüftdysplasie deutlich geringere Erfolgsaussichten und die Hüfttotalprothesenimplantation sollte erwogen werden [62]. Weiter kann bei vermuteter Beinlängendifferenz ein Röntgenbild beider Beine oder eine EOS-Beinaufnahme angefertigt werden.

**Figure 1:**
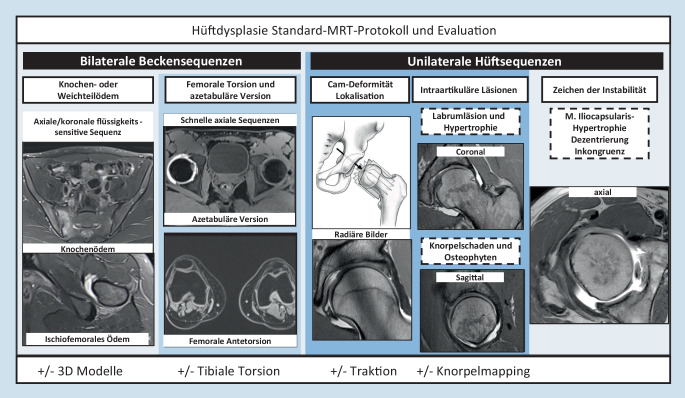

Die Schnittbildgebung (CT oder MRT) ist der Goldstandard zur Beurteilung der azetabulären und femoralen Morphologie, der assoziierten Deformitäten wie der Cam-Deformität und zur Messung der femoralen Antetorsion [21, 80]. Die MRT bei 1,5 T oder zunehmend bei 3 T hat einen hohen Stellenwert und wird in unserer Abteilung routinemäßig bei Patienten mit Hüftdysplasie angewendet. Initial wurde die MRT aufgrund der hohen Sensitivität zur Detektion von Labrum- und Knorpelschäden durchgeführt. Ein mögliches MRT-Protokoll der Hüfte bei Patienten, die für eine gelenkserhaltende Operation, z. B. im Rahmen einer Hüftdysplasie, evaluiert werden, wird in Abb. 1 dargestellt. Grundsätzlich unterscheiden wir beckenzentrierte und hüftzentrierte Sequenzen für unterschiedliche Fragestellungen [42].

**Figure 2:**
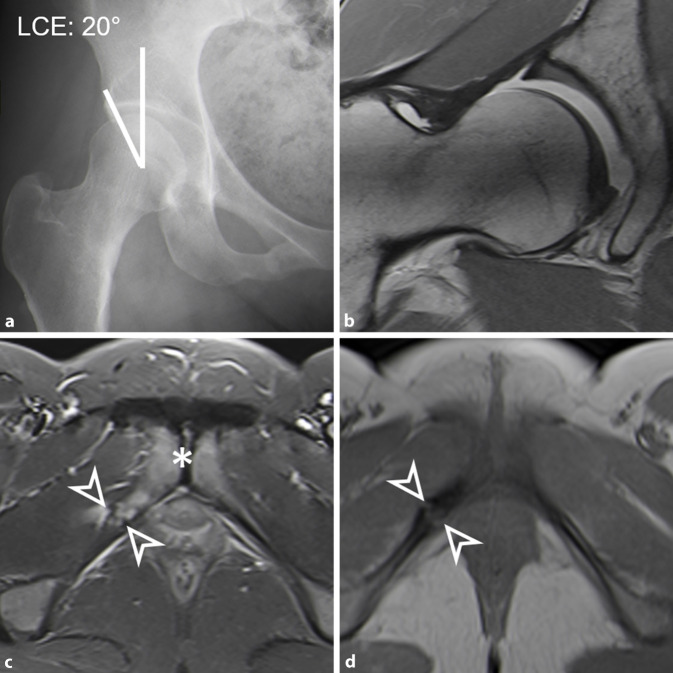

**Figure 3:**
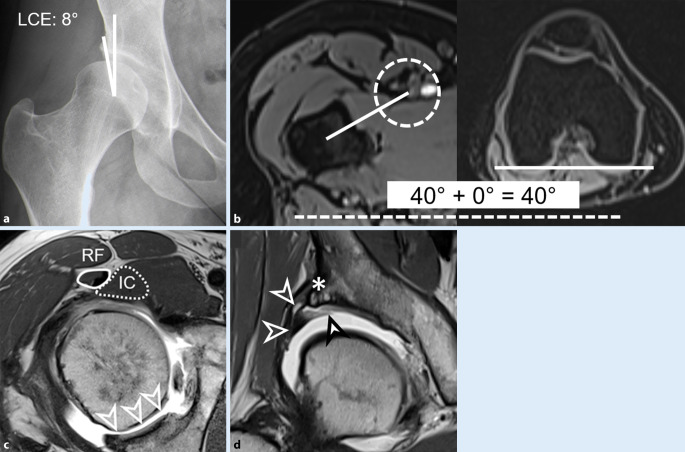

Sequenzen mit einem großen „field of view“ des gesamten Beckens (gesamtes Becken abgebildet) dienen einerseits der Detektion von ossären Stressreaktionen (Abb. 2c) oder entzündlichen Veränderungen des Gelenks und der periartikulären Strukturen (z. B. Symphyse, Iliosakralgelenk [15] oder ischiofemorales Ödem [78]) und andererseits der Erkennung von relevanten Nebendiagnosen im kleinen Becken oder in der Leiste [69] (z. B. inkarzerierte Leistenhernie oder Ovarialzyste). Hierfür werden flüssigkeitssensitive Sequenzen verwendet (fettgesättigte T2 TSE- oder TIRM‑/STIR Sequenzen) [42]. Schnelle axiale Sequenzen über Becken und Knie (z. B. dreidimensionale [3-D] T1w-Dixon-VIBE [„volumetric interpolated breath-hold examination“]) haben einen guten Kontrast zwischen Knochen und Weichteilen und können somit zur Messung der femoralen Antetorsion ([68]; Abb. 3b) und der azetabulären Version verwendet werden.

Hüftzentrierte Bilder (unilaterales Hüftgelenk abgebildet) dienen in erster Linie zur Detektion von chondrolabralen Schäden, wobei bevorzugt die direkte MR-Arthrographie oder ein natives Protokoll bei 3 T verwendet werden können. Dafür werden hochaufgelöste Bilder in den Standardorientierungen (koronal, axial und sagittal) akquiriert oder sie werden aus einer 3‑D-Sequenz rekonstruiert. Für die hüftzentrierten Sequenzen können entweder 2D-TSE/FSE-Sequenzen verwendet werden (direkt) oder es werden isotrope 3‑D-Sequenzen (z. B. 3D-T2w-TRUFI) akquiriert und mittels MPR in allen Ebenen rekonstruiert [40].

Zusätzlich zu den üblichen Standardsequenzen können radiäre Bilder zur Beurteilung der Cam-Deformität und von chondrolabralen Schäden akquiriert werden. Diese rotieren orthogonal um die Schenkelhalsachse und erlauben somit eine zirkumferenzielle Darstellung des Schenkelhals-Kopf-Übergangs. Alternativ können dafür axial oblique Sequenzen verwendet werden, die jedoch weniger sensitiv in der Detektion von Cam-Deformitäten sind [50].

Zusätzlich zu dem beschriebenen Protokoll in Abb. 1 können weitere Sequenzen zur Messung der tibialen Torsion, zur Erstellung von 3‑D-Modellen und spezifische hochauflösende Sequenzen für Knorpelmapping (z. B. „delayed gadolinium enhanced MRI of cartilage“ [dGEMRIC]) hinzugefügt werden. Eine Kombination mit Traktion [56] bei vorgängiger Arthrographie erscheint sinnvoll bei fortgeschrittenem Patientenalter.

Die CT wurde vorwiegend in amerikanischen Studien als Standard bezeichnet, im Rahmen der präoperativen Abklärung bei Patienten mit Hüftdysplasie [46, 80]. Im Vergleich zur MR-Arthrographie erlaubt die CT-Arthrographie ebenfalls die Detektion chondrolabraler Läsionen mit einer hohen diagnostischen Genauigkeit. Vorteil der CT ist die hohe Verfügbarkeit, kürzere Untersuchungszeit und die Erstellung von patientenspezifischen 3‑D-Modelle. Diese 3‑D-Modelle können zur PAO-Planung oder zur Impingement-Simulation verwendet werden, was vor allem bei Patienten mit erhöhter femoraler Antetorsion hilfreich sein kann [36]. Erste Studien haben erfolgreich MRT-basierte 3‑D-Modelle zu diesem Zweck verwendet [82, 83].

In den folgenden Abschnitten werden die radiologischen Parameter detailliert beschrieben.

## Die azetabuläre Version

Die Messung der azetabulären Version erfolgt auf axialen T1w-Bildern und kann analog zur Messung im CT erfolgen. Studien belegen die gute Korrelation zwischen CT und MRT bei Erwachsenen und pädiatrischen Patienten [17, 53]. Die azetabuläre Version wurde in einer aktuellen CT-Studie [61] von 1–3 Uhr gemessen: 8° bei 1 Uhr, 15° bei 2 Uhr und 21° bei 3 Uhr. Frühere Studien haben eine azetabuläre Version bei 3 Uhr zwischen 17 und 25° [27] und bis zu 27° für weibliche Patienten und 19° für männliche Patienten mit Hüftdysplasie beschrieben [34]. Neun mögliche Kombinationen der azetabulären Version und der femoralen Antetorsion wurden beschrieben (Tab. 2). Je nach Kombination können diese z. B. eine anteriore Instabilität verstärken, wenn eine erhöhte azetabuläre Version kombiniert mit einer femoralen Antetorsion vorliegt [34, 77].

**Table 2:** 

	*Erhöhte femorale Antetorsion*	*Normale femorale Antetorsion*	*Femorale Retrotorsion*
*Erhöhte azetabuläre Version*	**Posteriores FAI, erhöhte Innenrotation**	–	Mögliche Kompensation
*Normale azetabuläre Version*	–	**Normal 10–25°**	–
*Azetabuläre Retroversion*	Mögliche Kompensation	–	**Anteriores FAI, tiefe Innenrotation**

*FAI* femoroazetabuläres Impingement

Die azetabuläre Morphologie kann in anteriore, laterale und posteriore Dysplasie eingeteilt werden mithilfe des sogenannten anterioren und posterioren Sektorwinkel (AASA [„anterior acetabular sector angle“] und PASA). Die Summe von AASA und PASA ergibt den HASA („horizontal acetabular sector angle“).

In einer früheren Studie wurde beschrieben, dass bis zu 17 % der Patienten mit Hüftdysplasie eine azetabuläre Retroversion aufweisen [37], während Tönnis sogar bei 29 % der Patienten mit Hüftdysplasie eine azetabuläre Version < 10° festgestellt hat. Nachfolgende Studien haben eine tiefere Häufigkeit angegeben [43].

## Proximale femorale Deformitäten

Kombinierte femorale Deformitäten sind bei der Hüftdysplasie häufig, typisch ist eine ellipsoide Form des Femurkopfes [63]. Eine Cam-Deformität kann bei bis zu 86 % der Patienten vorliegen [80] (definiert als reduzierter Offset oder Alphawinkel > 55°). Valgusdeformitäten wurden bei 44 % beschrieben [6], während nur bis zu 4 % eine Coxa vara aufweisen. Eine Hüftdysplasie kombiniert mit erhöhter femoraler Antetorsion kann bei bis zu 52 % der Patienten vorliegen [80] (definiert als > 20°) (Abb. 3). Weiter wurde eine erhöhte femorale Antetorsion (definiert als > 35°) bei 8–23 % beschrieben [34]. Die femorale Antetorsion ist abhängig vom Geschlecht und beträgt 27° (Frauen) und 19° (Männer), gemessen bei Patienten mit Hüftschmerzen mit der Murphy-Methode [34]. Eine femorale Antetorsion unter 0° (= femorale Retrotorsion) tritt selten in Kombination mit einer Hüftdysplasie auf. Bei erhöhter femoraler Antetorsion > 35° kann ein posteriores intra- und extraartikuläres (ischiofemorales) Impingement zwischen dem Trochanter minor und dem Tuber ischiadicum vor allem bei Extension und Außenrotation der Hüfte auftreten [36]. Das knöcherne Anschlagen zwischen Ischium und Trochanter minor/major führt möglicherweise zu einer anterioren Translation des Femurkopfs und damit zu einer dynamischen Instabilität [36].

Die Cam-Deformität wird klassischerweise mittels Alphawinkel quantifiziert, wie von Nötzli initial beschrieben [47]. Die MRT mit radiären Bildern erlaubt die detaillierte Beschreibung und Ausdehnung der Cam-Deformität [55]. Weiter kann eine „offset ratio“ unter 0,17 verwendet werden zur Diagnostik der Cam-Deformität [80]. Die Cam-Deformität ist Folge der aufgehobenen Schenkelhalstaillierung. Typischerweise ist der Femurkopf-Schenkelhals-Übergang bei der Hüftdysplasie ellipsoid geformt [63]. Die Cam-Deformität ist meist anterior-superior oder anterior-lateral lokalisiert [1].

Die Beschreibung des Schweregrades und der Ausdehnung der Cam-Deformität erfolgt mittels CT/MRT unter Verwendung des Zifferblattsystems [42] (z. B. 3 Uhr entspricht anterior). Diese wurde mehrfach beschrieben und gehört bereits zur Standarddiagnostik, deswegen verzichten wir hier auf weitere Ausführungen [25].

Valgusdeformitäten werden meistens auf a.-p. Röntgenbildern diagnostiziert mittels CCD-Winkel. Meistens wurde eine Valgusdeformität als CCD-Winkel > 139° definiert [6, 77], obwohl es unterschiedliche Definitionen gibt.

## Femorale Antetorsion

Konventionelle Röntgenbilder (wie die Dunn-Rippstein Aufnahme) sind nicht verlässlich zur Bestimmung der femoralen Antetorsion [29] und erfordert eine Diagnostik mittels Schnittbildern. Die femorale Antetorsion wurde zwar bereits vor Jahrzehnten untersucht, u. a. von Tönnis und Kollegen in den 1990er-Jahren [76, 77]. Jedoch wurde die femorale Antetorsion selten auf Schnittbildern (CT oder MRT) quantifiziert [29]. Aufgrund der zunehmenden Wichtigkeit wird die femorale Antetorsion detailliert behandelt. Die Diagnostik kann mittels CT oder MRT erfolgen, beide Modalitäten weisen eine gute Reproduzierbarkeit und Reliabilität auf [17, 21]. Die CT wird aufgrund der einfacheren Verfügbarkeit vielfach verwendet, jedoch konnte in mehreren Studien gezeigt werden, dass mittels Akquisition von schnellen MRT-Bildern des Beckens und des Knies (für femorale Kondylenebene) eine akkurate Bestimmung der femoralen Torsion verlässlich möglich ist. Die Messung mittels MRT ist besonders für junge Frauen im gebärfähigen Altern sinnvoll, sie hat den Vorteil, auf ionisierende Strahlung verzichten zu können [21].

**Figure 4:**
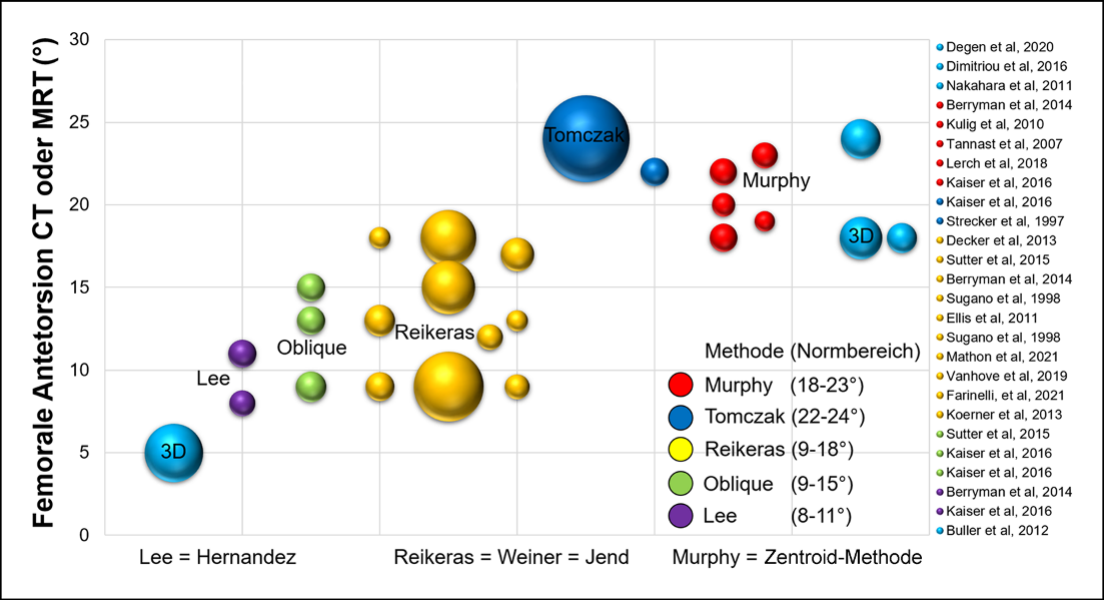

Bei der Messung der femoralen Antetorsion sollten die verschiedenen Messmethoden [57] und entsprechende Normalwerte berücksichtigt werden. Die Messmethoden gehen vom Femurkopfzentrum aus, und unterscheiden sich in der Wahl des zweiten proximalen Referenzpunkts [57]. Je weiter distal dieser Referenzpunkt gesetzt wird, desto höher fallen die gemessenen Werte aus. Diese Unterschiede können beim selben Patienten bis zu 20° betragen [57]. Diese Unterschiede sind vor allem bei Patienten mit erhöhter femoraler Antetorsion und bei Valgusdeformitäten zu beachten [59]. Die Normwerte der jeweiligen Messmethoden sollten beachtet werden (Tab. 3). Für die Murphy-Methode (Abb. 4) wurden Normalwerte von 18–23° beschrieben [2, 22], hingegen wurden für die Reikeras-Methode Normalwerte von 9–18° beschrieben [9, 26]. Aufgrund der verschiedenen Messmethoden besteht die Gefahr von falsch hohen oder falsch tiefen Messwerten, was zu Fehldiagnosen führen kann, sofern keine standardisierte Messmethode verwendet wird.

**Table 3:** 

Autor (Jahr)	Messmethode	Beschreibung
Lee et al. 2006 [57]	**CT, axiale Schichten**	*Lee-Methode: *Linie zur Verbindung vom Femurkopfzentrum und der Mitte des Schenkelhalses auf dem ersten axialen Schnitt, auf dem eine Verbindung zwischen Schenkelhals und Trochanter major sichtbar ist
Reikerås et al. 1983 [57]	**CT, axiale Schichten**	*Reikeras-Methode: *Linie zur Verbindung vom Femurkopfzentrum und der Mitte des Schenkelhalses auf einem axialen Schnitt, auf welchem der anteriore und der posteriore Kortex des Schenkelhalses parallel sind
Tomczak et al. 1997 [57]	**MRT, axiale Schichten**	*Tomczak-Methode: *Linie zur Verbindung vom Femurkopfzentrum und dem Zentrum des Trochanter major an der Basis des Schenkelhalses
Murphy et al. 1987 [57]	**CT, axiale Schichten**	*Murphy-Methode: *Linie zur Verbindung vom Femurkopfzentrum und der Mitte des Zentroids auf einem axialen Schnitt am Oberrand des Trochanter minor
Jarret et al. 2010 [57]	CT, axial oblique Schichten	*Jarret-Methode: *Linie zur Verbindung vom Femurkopfzentrum und der Mitte des Schenkelhalses auf einer schrägen Ebene in der Schenkelhalsachse

Eine aktuelle Studie hat bei Patienten mit erhöhter femoraler Antetorsion > 35° ein posteriores extraartikuläres ischiofemorales Impingement festgestellt [36]. Dieses kann zwischen dem Tuber ischiadicum und dem Trochanter major und/oder dem Trochanter minor auftreten. Dafür wurden CT-basierte 3‑D-Modelle ausgewertet und ein ossäres Impingement simuliert. Weiter kann zur Diagnostik des ischiofemoralen Impingements mittels flüssigkeitssensitiven Sequenzen das ischiofemorale Ödem (im M. quadratus femoris) diagnostiziert werden und ein reduzierter ischiofemoraler Abstand gemessen werden [4, 78].

Aufgrund der verschiedenen Messmethoden der femoralen Antetorsion besteht die Gefahr von falschen Messwerten

**Figure 5:**
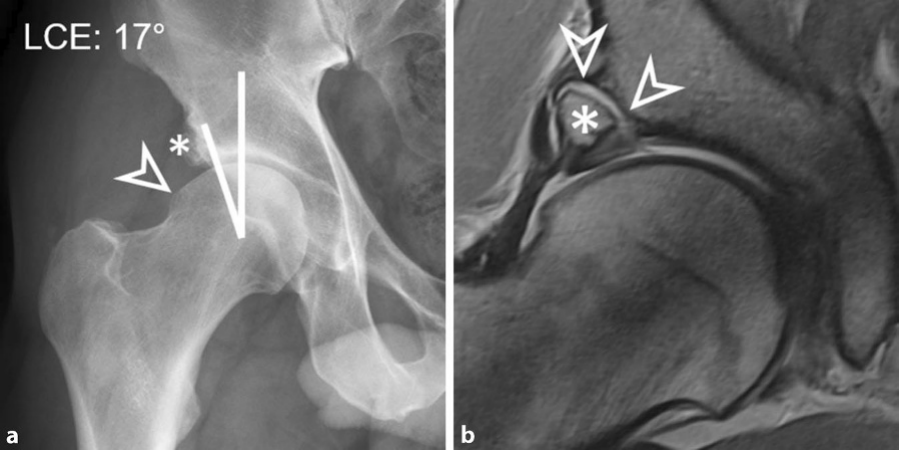

Typischerweise wurde bei Patienten mit einer Hüftdysplasie eine anteriore Labrumläsion und eine Adaption des Labrums mit Hypertrophie und mukoider Degeneration beobachtet [42]. Je nach Schweregrad der Minderüberdachung bzw. der bereits bestehenden Gelenkdegeneration kann es auch zu paralabralen Ganglien, Knochenzysten oder einer Knorpeldelamination führen (Abb. 3). Die Instabilität kann in Kombination mit der Cam-Deformität auch bei Patienten mit Hüftdysplasie zu einer Sressfraktur am Pfannenrand führen (Os acetabuli) (Abb. 5). Osteophyten treten im Vergleich zum Impingement-Patienten tendenziell später auf [19]. Eine Vielzahl von intraoperativen Klassifikationen existieren für chondrolabrale Läsionen, die meisten basierend auf intraoperativen Bildern [81]. Zudem wurden auch mehrere MRT-Klassifikationen für Labrumläsionen beschrieben [3, 7, 55], mit denen eine hohe Sensitivität von über 90 % zur Detektion von Labrumläsionen mittels MR-Arthrographie der Hüfte erzielt wurde [14].

Die typische Knorpelläsion ist eine oberflächliche oder entspricht einer „Inside-out“-Läsion bei der es zu einer teppichartigen Delamination des azetabulären Knorpels von zentral nach peripher mit Riss bis ins Labrum kommt. Im Gegensatz zum Cam-FAI, wo der Knorpel sich aufgrund der repetitiven Scherkräfte von der chondrolabralen Übergangszone aus nach zentral ablöst. Die MRT-Diagnostik von Knorpelschäden ist aufgrund der dünnen Knorpelschichten, die einander direkt anliegen, schwierig. Die MR-Arthrographie ermöglicht die Detektion von Knorpelschäden mit höherer diagnostischer Genauigkeit als das native MRT, und hat den Vorteil, dass sie bei Bedarf mit der Applikation von Traktion kombiniert werden kann, um insbesondere die Darstellung der Knorpeldelaminationen zu verbessern [54, 67].

Außerdem ermöglicht die MRT die Beurteilung von subtilen Zeichen der Hüftinstabilität. Diese Zeichen sind einerseits die Hypertrophie des M. iliocapsularis im Vergleich zum M. rectus femoris auf axialen Schichten [18] sowie die anteriore Dezentrierung oder Subluxation des Femurkopfs (Abb. 3c). Letzteres wurde in der MR-Arthrographie beschrieben und ist gemäß aktueller Literatur ein nützliches Zeichen zur Beurteilung der Instabilität bei Patienten mit grenzwertiger Hüftdysplasie [84].

## Beckenkippung, „pelvic incidence“ und „Hip-spine“-Syndrom

Die Beckenkippung ist ein dynamischer Parameter, welcher die azetabuläre Überdachung beeinflussen kann. Deshalb wurde beschrieben, dass die Beckenkippung zu beachten sei bei geplanter PAO. Leider existieren verschiedene Definitionen der Beckenkippung (u. a. „pelvic tilt“), was zu Missverständnissen führen kann. Grundsätzlich kann gesagt werden, dass eine anteriore Beckenkippung die anteriore azetabuläre Überdachung erhöht. Eine signifikante Korrelation zwischen „pelvic tilt“ und azetabulärer Version (CT-Messung) wurde bei 100 Patienten mit Hüftarthrose aufgrund einer Hüftdysplasie (*p* < 0,001; r = 0,389) beschrieben [49]. Eine kürzlich erschienene Studie zeigte eine bessere Korrelation (*p* < 0,001; r = 0,58) zwischen „pelvic tilt“ und azetabulärer Version (CT-Messung) [35]. Nach bilateraler PAO wurde eine leichte Reduktion der anterioren Beckenkippung beschrieben, jedoch erfolgten die Messungen auf dem Röntgenbild [8]. Außerdem ist die Beckenkippung abhängig von der Körperposition und variiert zwischen stehender und sitzender Position.

Für die Wirbelsäulenchirurgie werden drei Parameter der sagittalen Balance verwendet: „pelvic tilt“, „sacral slope“ und „pelvic incidence“ [30, 51]. Kürzlich wurden bei Patienten mit Hüftdysplasie diese drei Parameter folgendermaßen beschrieben : „pelvic tilt“ von 10°, „pelvic incidence“ von 55° und „sacral slope“ von 44°, gemessen beim liegenden Patienten im CT [35]. „Pelvic incidence“ ist ein statischer Parameter, der sich nicht verändert zwischen stehender und sitzender Position [31]. Bei Patienten mit Hüftdysplasie und erhöhter azetabulärer Version > 25° (CT) wurde eine signifikant höhere „pelvic incidence“ von 57° gefunden im Vergleich zu Patienten mit azetabulärer Retroversion („pelvic incidence“ von 42°) [35]. Weiter wurde eine tiefere „pelvic incidence“ bei Hüft-Impingement-Patienten gefunden [51]. In einer anderen Übersichtsarbeit wurde „pelvic tilt“ als „spinopelvic tilt“ bezeichnet [23]. Für die anteriore Beckenkippung wurde der sogenannte „anterior pelvic plane tilt“ gemessen [23]. In einer kürzlich publizierten Übersichtsarbeit wurde das lumbo-pelvine-azetabuläre Alignment detailliert beschrieben [20]. Dabei wurden u. a. sowohl die verschiedenen radiologischen Parameter beschrieben als auch vier verschiedene Typen der Beckenform und deren sagittales (lumbopelvines) Alignment [20]. Eine Beckenform Typ D (erhöhte pelvic incidence) kann die azetabuläre Anteversion verstärken, was bei Patienten mit Hüftdysplasie wichtig sein könnte.

Initial wurden Rückenschmerzen, welche mit einer anterioren Beckenkippung und Hyperlordose assoziiert waren, als sogenanntes „Hip-spine“-Syndrom beschrieben [48]. Dieses „Hip-spine“-Syndrom wurde bei Kindern mit kongenitaler Hüftluxation beschrieben [39]. Kürzlich hat eine biomechanische Studie eine höhere Belastung der lumbalen Facettengelenke bei Patienten mit Hüftschmerzen und posteriorem ischiofemoralem Impingement beschrieben [16]. Möglicherweise gibt es funktionelle Kompensationsmechanismen, sodass eine verminderte anteriore azetabuläre Überdachung durch anteriore Beckenkippung und Hyperlordose kompensiert wird. Umgekehrt kann möglicherweise ein Cam-Typ Hüft-Impingement durch posteriore Beckenkippung kompensiert werden [51].

## Ergebnisse und Diskussion

Mehrere Langzeitstudien haben die Ergebnisse nach PAO evaluiert. Diese Studien wurden in einer Grafik in einer früheren Übersichtsarbeit zusammengefasst [32]. Für die erste Serie von 75 Hüften, welche mittels der PAO nach Ganz therapiert wurden, sind die Resultate nach 20 und 30 Jahren beschrieben worden [33, 62]. Bei dieser Gruppe hatten nach 20 Jahren 40 % der Hüften eine Hüfttotalprothese, jedoch war die Gruppe bezüglich vorbestehender Arthrose und Voroperationen sehr heterogen [62]. Dabei war für ein schlechtes Langzeitüberleben der Hüftgelenke folgender Faktor maßgebend: eine vorbestehende Arthrose, wobei Hüften mit einem Arthrosegrad > 1 nach Tönnis zu 88 % nach 20 Jahren eine Hüfttotalprothese hatten. Zudem waren weitere klinische Faktoren (erhöhtes Alter, vorbestehende starke Schmerzen und Hinken) assoziiert mit verfrühtem endoprothetischem Ersatz. Weitere radiologische Faktoren, welche mit einem schlechteren Langzeitergebnis assoziiert waren, sind in Tab. 4 zusammengefasst.

**Table 4:** 

Prädiktoren für eine Hüfttotalprothese	Risikoquantifizierung
Postoperativer Extrusionsindex	11 % Risikozunahme pro Prozent weniger
„Lateral center edge“-Winkel unter 0° präoperativ	4,7fach erhöhtes Risiko nach 9 Jahren
Exzessive laterale und proximale Dislokation des Femurkopfes präoperativ	4fach erhöhtes Risiko nach 9 Jahren
Postoperative Breite der Sklerosezone < 2,5 cm	6fach erhöhtes Risiko nach 9 Jahren
Vorhandenes Os acetabuli	3,6fach erhöhtes Risiko nach 9 Jahren
Azetabuläre Anteversion unter 10°	4,3fach erhöhtes Risiko nach 9 Jahren

Eine kürzlich erschiene systematische Analyse von Studien zeigte, dass das präoperative Alter und der präoperative Arthrosegrad die stärksten Einflussfaktoren für das Langzeitergebnis (10 und 20 Jahre) darstellen [70]. Diese Studien zeigten, dass die präoperative Bildgebung bei Patienten mit Hüftdysplasie vor allem zur Evaluation von Knorpelschäden prognostisch wichtig ist.

**Figure 6:**
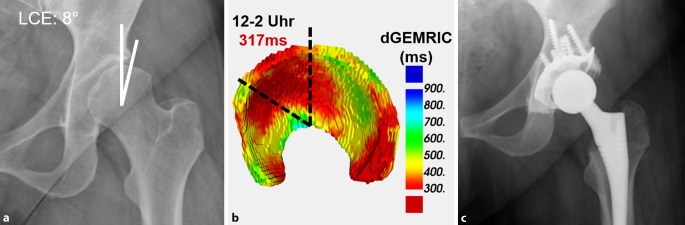

Für 1,5 T wurde mittels dGEMRIC ein Prädiktor für den Verlauf nach PAO beschrieben [24]. Nach einer mittleren Untersuchungszeit von 32 Monaten war ein tieferer anteriorer präoperativer dGEMRIC-Werte als Hinweis auf eine fortgeschrittene biochemische Knorpeldegeneration mit einem schlechteren Outcome assoziiert [24]. Neue Entwicklungen im Bereich der Sequenztechnik und in der Bildverarbeitung mittels neuronaler Netzwerke („machine learning“ oder „deep learning“) ermöglichen die Erstellung von 3‑D-Knorpelmodellen für eine frühe, objektive Quantifizierung von Knorpelschäden ([52, 58]; Abb. 6).

## Fazit für die Praxis

- Grundlage der präoperativen Bildgebung vor hüftgelenkerhaltenden Eingriffen sind die Beckenübersichtsaufnahme zur Beurteilung der azetabulären Überdachung und Version sowie die initiale Beurteilung des Arthrosegrades.
- Die MRT sollte beckenzentrierte, flüssigkeitssensitive Sequenzen zur Detektion von Begleitpathologien, axiale Sequenzen zur Bestimmung der Femurtorsion sowie hüftzentrierte, multiplanare Sequenzen zur Detektion chondrolabraler Schäden beinhalten.
- Die direkte MR-Arthrographie der Hüfte oder alternativ die native 3 T MRT sind die Methoden der Wahl zur Detektion von chondrolabralen Schäden.
- Die azetabuläre Morphologie kann in anteriore, laterale und posteriore Dysplasie eingeteilt werden. Kombinierte Deformitäten sind bei der Hüftdysplasie häufig.
- Radiologische prädiktive Faktoren für ein schlechteres Outcome wurden beschrieben.
- Bei der präoperativen Bildgebung sind sowohl die azetabuläre Morphologie als auch die femoralen Deformitäten, u. a. die femorale Antetorsion und cam Deformitäten zu berücksichtigen.

## Abkürzungen

AASA: „Anterior acetabular sector angle“
AI: Azetabulärer Index
CCD-Winkel: Centrum-Collum-Diaphysen-Winkel
dGEMRIC: „Delayed gadolinium enhanced MRI of cartilage“
FAI: Femoroazetabuläres Impingement
FEAR: „Femoro-epiphyseal acetabular roof“
FSE: „Fast spin echo“
HASA: „Horizontal acetabular sector angle“
LCE: „Lateral center edge“
MPR: „Multi planar reconstruction“
PAO: Periazetabuläre Osteotomie
PASA: „Posterior acetabular sector angle“
STIR: „Short tau inversion recovery“
TIRM: „Turbo-inversion recovery magnitude“
TRUFI: „True fast imaging with steady precession“
TSE: Turbo Spin Echo
VIBE: „Volumetric interpolated breath-hold examination“
